# Supplementary material for: SARS-CoV-2 genomic surveillance in wastewater as a model for monitoring evolution of endemic viruses
Source: Nat Commun. 2023 Oct 10;14:6325. doi: 10.1038/s41467-023-41369-5 (PMC10564906; doi:10.1038/s41467-023-41369-5)
Supplement: Supplementary file 1 — Supplementary Information [file 41467_2023_41369_MOESM1_ESM.pdf]

Supplementary figure S1

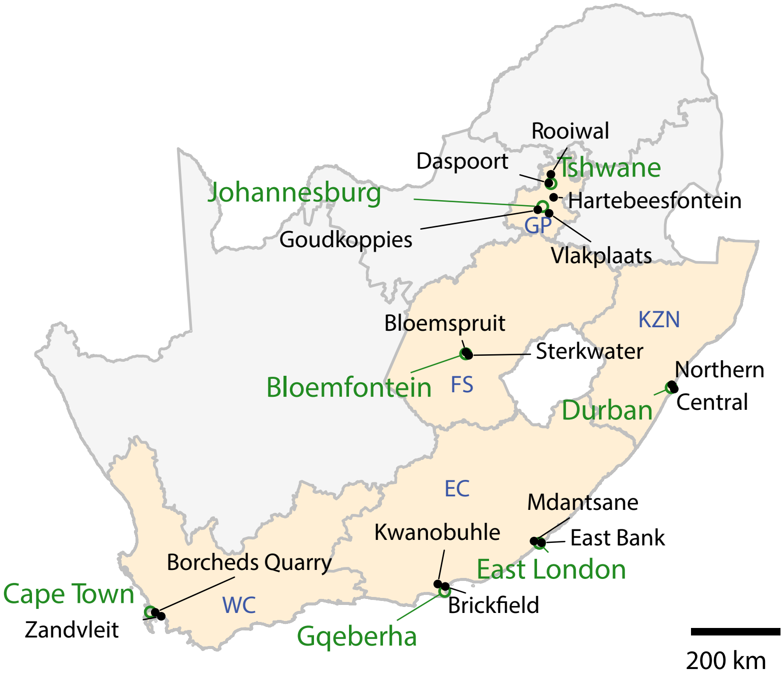

A map showing the distribution of wastewater treatment plants in South Africa

Supplementary figure S2

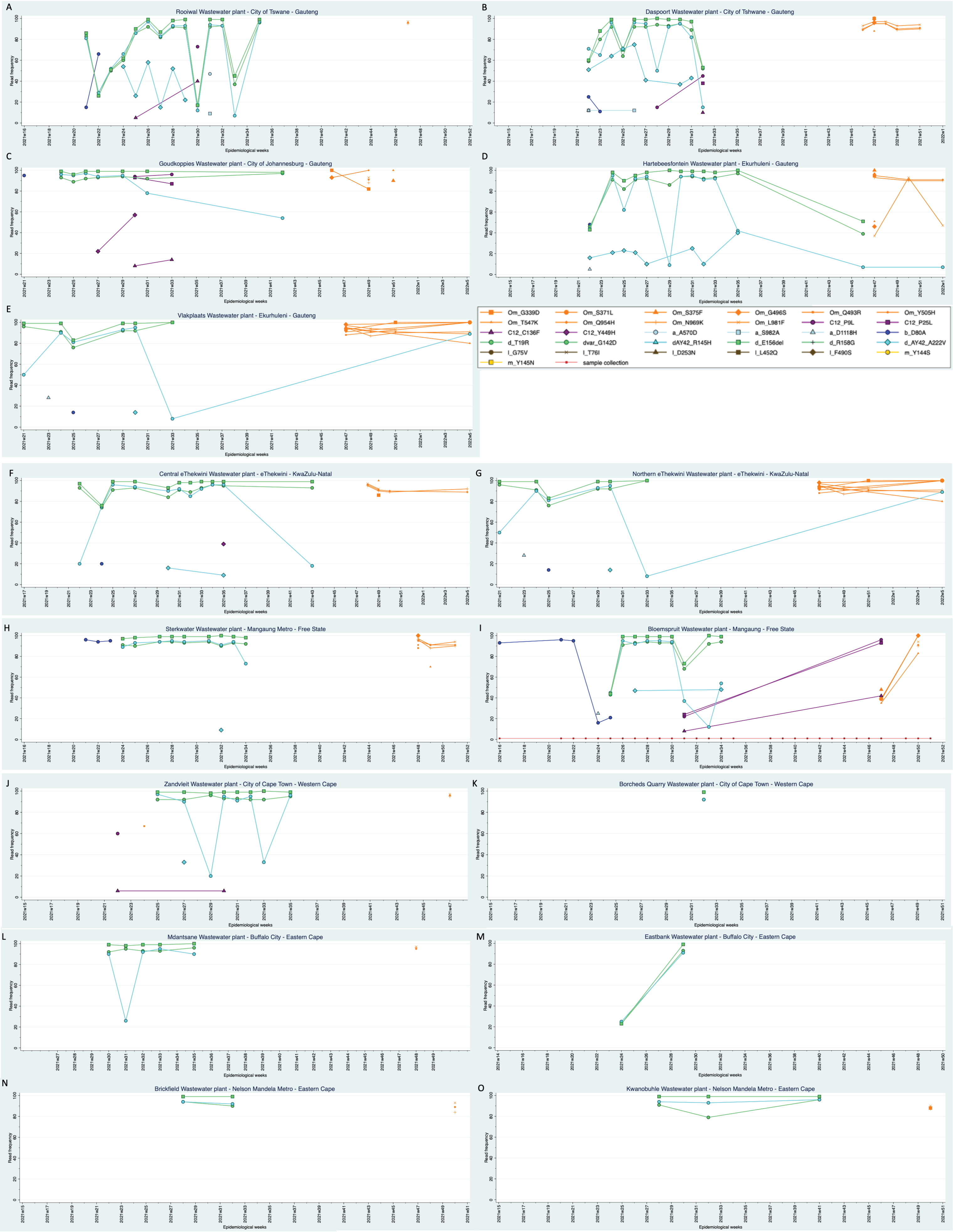

Signature mutation analysis of Variants of concern (VOC) and Variants of interest (VOI)

Supplementary figures

Figure S1. A map showing the distribution of wastewater treatment plants in South Africa for the study. Provinces are denoted in Blue, using the abbreviations GP=Gauteng Province, KZN=KwaZulu-Natal, FS=Free State, EC=Eastern Cape, and WC=Western Cape.

Figure S2. Signature mutation analysis of Variants of concern (VOC) and Variants of interest (VOI). The Y axis shows the read frequencies of each mutation, and X axis shows the epidemiological week when the samples were collected. The red line with dots shows the time-point analysed. Different shapes and colours of signature mutations were shown in the key. A-E: Gauteng province sites. F-G: KwaZulu-Natal province. H-I: Free State province. J-K: Western Cape province. L-O: Eastern Cape province.

Table S1. Characteristics of wastewater treatment facilities and samples submitted for SARS-CoV-2 sequencing from the below sites, between April 2021- Jan 2022.

Province	Metro or District	Plant name	Population size served by the facility	Genomic testing	
				Epidemiological week when sequencing started in 2021	# samples submitted for sequencing
Eastern Cape	Buffalo City Metro	East Bank	141000	15	16
		Mdantsane	112900	25	19
	Nelson Mandela Metro	Brickfield	40000	15	7
		KwaNobuhle	100320	15	7
Free State	Mangaung	Sterkwater	200000	16	28
		Bloemspruit	350000	16	30
Gauteng	Ekurhuleni Metro	Daveyton	100000	20	5
		Hartebeesfontain	100000	14	27
		Vlakplaats	200000	21	19
	Johannesburg Metro	Northern	1200000	14	6
		Goudkoppies	500000	21	20
	Tshwane Metro	Rooiwal	unknown	17	29
		Daspoort	unknown	14	30
Kwazulu-Natal	eThekwini Metro	Northern	316425	17	25
		Central	350000	17	27
Western Cape	City of Cape Town Metro	Borcherd's Quarry	380000	15	6
		Zandvliet	460000	15	24
Total					325

Table S2. Illustration of amino acid variations in samples, produced by the Galaxy pipeline.

A	B	C	D	E	F	G
**Sample**	**QC filter**	**Number of reads**	**Mutation effect**	**GENE**	**Mutation**	**Mutation frequency**
21-0128	PASS	4073	NON_SYNONYMOUS_CODING	ORF1ab	E41K	92
21-0128	PASS	12106	NON_SYNONYMOUS_CODING	S	L1203F	96
21-0128	min_af_0.1Xmin_ dp_5Xmin_dp_ alt_10	12234	FRAME_SHIFT	S	V1228	1
21-0128	PASS	13569	NON_SYNONYMOUS_CODING	ORF3a	T190I	88
21-0128	PASS	1536	NON_SYNONYMOUS_CODING	E	P71L	97
21-0128	min_af_0.1Xmin_ dp_5Xmin_dp_ alt_10	1339	NON_SYNONYMOUS_CODING	M	I8S	1

A shows sample ID. **B** is the quality indicator. Filter value 'PASS' are samples with a minimum of 10 reads and read frequency greater than 10. **C** is the number of reads produced for each sample. **D** is the effect of the mutation detected in the gene. **E** is the name of the gene where mutation occurred. **F** is the mutation detected. **G** is the proportion of the mutant cells in a sample or population.

Table S3: List of signature mutations which was used to identify VOC and VOI present in wastewater samples

**Omicron**	**Alpha**	**Beta**	**Delta**	**C.1.2**	**Gamma**	**Lambda**	**Mu**
G339D	A570D	D80A	T19R	P9L	T20N	G75V	Y144S
S371L	S982A		R145H	P25L	P26S	T76I	Y145N
S375F	D1118H		E156del	C136F	T1027I	D253N	
Q493R			R158G	Y449H		L452Q	
G496S			A222V			F490S	
Y505H							
T547K							
N856K							
Q954H							
N969K							
L981F							

Table S4: Amino acid mutations detected in wastewater samples that were commonly detected in clinical cases

**Mutation**	**Date first detected**	**Date last detected**	**Number of samples detected in Wastewater**	**Prevalence in wastewater in South Africa**
P9L	May, 2021	November, 2021	8	2,46
T19R	May, 2021	November, 2021	120	36,92
T19I	January, 2022	January, 2022	6	1,85
L24-	January, 2022	January, 2022	6	1,85
P25-	January, 2022	January, 2022	6	1,85
P26-	January, 2022	January, 2022	6	1,85
A27S	January, 2022	January, 2022	6	1,85
P25L	July, 2021	November, 2021	5	1,54
A67V	April, 2021	January, 2022	20	6,15
T95I	May, 2021	January, 2022	34	10,46
C136F	May, 2021	November, 2021	9	2,77
G142D	May, 2021	January, 2022	125	38,46
Y144-	21-May	21-Nov	20	6,15
E156-	May, 2021	November, 2021	120	36,92
F157-	May, 2021	November, 2021	120	36,92
R158G	May, 2021	November, 2021	120	36,92
R190S	July, 2021	November, 2021	9	2,77
N211-	August, 2021	January, 2022	20	6,15
L212I	August, 2021	January, 2022	20	6,15
V213G	January, 2022	January, 2022	4	1,23
D215G	April, 2021	November, 2021	23	7,08
A222V	June, 2021	August, 2021	33	10,15
L242-	June, 2021	January, 2022	22	6,77
A243-	June, 2021	January, 2022	35	10,77
G339D	November, 2021	January, 2022	13	4,00
S371F	November, 2021	January, 2022	8	2,46
S373P	November, 2021	January, 2022	7	2,15
S375F	November, 2021	January, 2022	7	2,15
K417N	May, 2021	January, 2022	20	6,15
K417T	May, 2021	November, 2021	13	4,00
N440K	June, 2021	January, 2022	8	2,46
G446S	November, 2021	January, 2022	7	2,15
L452R	May, 2021	November, 2021	75	23,08
S477N	November, 2021	January, 2022	10	3,08
T478K	May, 2021	January, 2022	86	26,46
E484K	May, 2021	July, 2021	16	4,92
E484A	June, 2021	January, 2022	18	5,54
Q493R	June, 2021	January, 2022	11	3,38

G496S	November, 2021	January, 2022	9	2,77
Q498R	July, 2021	January, 2022	12	3,69
N501Y	May, 2021	January, 2022	23	7,08
Y505H	September, 2021	January, 2022	10	3,08
T547K	November, 2021	January, 2022	9	2,77
D614G	April, 2021	January, 2022	108	33,23
H655Y	May, 2021	January, 2022	46	14,15
N679K	June, 2021	January, 2022	50	15,38
P681H	May, 2021	January, 2022	42	12,92
P681R	May, 2021	December, 2021	122	37,54
A701V	April, 2021	December, 2021	35	10,77
T716I	June, 2021	October, 2021	20	6,15
N764K	April, 2021	January, 2022	35	10,77
D796Y	November, 2021	January, 2022	34	10,46
D950N	May, 2021	December, 2021	122	37,54
Q954H	November, 2021	January, 2022	34	10,46
N969K	November, 2021	January, 2022	34	10,46
L981F	November, 2021	January, 2022	28	8,62
S982A	June, 2021	August, 2021	5	1,54
D1118H	June, 2021	June, 2021	5	1,54
