# Supplementary material for: SARS-CoV-2 genomic surveillance in wastewater as a model for monitoring evolution of endemic viruses
Source: Nat Commun. 2023 Oct 10;14:6325. doi: 10.1038/s41467-023-41369-5 (PMC10564906; doi:10.1038/s41467-023-41369-5)
Supplement: Supplementary file 3 — Reporting Summary [file 41467_2023_41369_MOESM3_ESM.pdf]

nature portfolio

Corresponding author(s): Mukhlid Yousif

Last updated by author(s): Aug 18, 2023

Reporting Summary

Nature Portfolio wishes to improve the reproducibility of the work that we publish. This form provides structure for consistency and transparency in reporting. For further information on Nature Portfolio policies, see our Editorial Policies and the Editorial Policy Checklist.

Statistics

For all statistical analyses, confirm that the following items are present in the figure legend, table legend, main text, or Methods section.

n/a	Confirmed	
☐	☒	The exact sample size (*n*) for each experimental group/condition, given as a discrete number and unit of measurement
☒	☐	A statement on whether measurements were taken from distinct samples or whether the same sample was measured repeatedly
☒	☐	The statistical test(s) used AND whether they are one- or two-sided<br>*Only common tests should be described solely by name; describe more complex techniques in the Methods section.*
☒	☐	A description of all covariates tested
☒	☐	A description of any assumptions or corrections, such as tests of normality and adjustment for multiple comparisons
☒	☐	A full description of the statistical parameters including central tendency (e.g. means) or other basic estimates (e.g. regression coefficient) AND variation (e.g. standard deviation) or associated estimates of uncertainty (e.g. confidence intervals)
☒	☐	For null hypothesis testing, the test statistic (e.g. *F*, *t*, *r*) with confidence intervals, effect sizes, degrees of freedom and *P* value noted<br>*Give P values as exact values whenever suitable.*
☒	☐	For Bayesian analysis, information on the choice of priors and Markov chain Monte Carlo settings
☒	☐	For hierarchical and complex designs, identification of the appropriate level for tests and full reporting of outcomes
☒	☐	Estimates of effect sizes (e.g. Cohen's *d*, Pearson's *r*), indicating how they were calculated

Our web collection on statistics for biologists contains articles on many of the points above.

Software and code

Policy information about availability of computer code

Data collection	Metadata for wastewater samples were collected in forms and captured in Microsoft Excel (2016).
Data analysis	All data analysis and visualization of wastewater genomic sequencing data was performed using the following:<br>1. Exatype v4.2.0-dev20230731 available at (https://sars-cov-2.exatype.com).<br>2. Bioinformatics pipeline on Galaxy, version 0.7.17.1. The pipeline uses BWA mem (Galaxy Version 0.7.17.1), samtools (Galaxy Version 1.9), iVar (Galaxy Version 1.2.2) and LoFreq (Galaxy Version 2.1.5).<br>3. Freyja (v1.3.10) hosted publicly on github (https://github.com/andersen-lab/Freyja) and is available under a BSD-2-Clause License (doi:10.5281/zenodo.6954863, version 1.3.10). Freyja is accessible as a package via bioconda (https://bioconda.github.io/recipes/freyja/README.html).<br>4. Custom R (version 4.2.0) and Python scripts (v 3.7.12) available in the project github repository (https://github.com/setshabaTaukobong/Heatmap-Matrix) and (https://github.com/setshabaTaukobong/Mutational-Dotplot).

For manuscripts utilizing custom algorithms or software that are central to the research but not yet described in published literature, software must be made available to editors and reviewers. We strongly encourage code deposition in a community repository (e.g. GitHub). See the Nature Portfolio guidelines for submitting code & software for further information.

Data

Policy information about availability of data

All manuscripts must include a data availability statement. This statement should provide the following information, where applicable:

- Accession codes, unique identifiers, or web links for publicly available datasets
- A description of any restrictions on data availability
- For clinical datasets or third party data, please ensure that the statement adheres to our policy

Data (raw sequence reads) have been deposited to NCBI with accession number (PRJNA941107), and can be found in the link: https://www.ncbi.nlm.nih.gov/bioproject/?term=PRJNA941107.
The following database have been used in the study:
1. The Global Initiative on Sharing All Influenza Data (GISAID), available at: https://gisaid.org/
2. Pango lineages: latest epidemiological lineages of SARS-CoV-2, available at: https://cov-lineages.org/
3. National Library of Medicine, National Center for Biotechnology Information (NCBI), accession number: NC_045512.2
4. Outbreak.info: a tool to explore the COVID-19 and SARS-CoV-2 data with variant surveillance reports, data on cases and deaths, and a standardized, searchable research library, available at: https://outbreak.info/

Research involving human participants, their data, or biological material

Policy information about studies with human participants or human data. See also policy information about sex, gender (identity/presentation), and sexual orientation and race, ethnicity and racism.

Reporting on sex and gender	Not applicable, the study used wastewater samples.
Reporting on race, ethnicity, or other socially relevant groupings	Not applicable, the study used wastewater samples.
Population characteristics	Not applicable, the study used wastewater samples.
Recruitment	Not applicable, the study used wastewater samples.
Ethics oversight	The study did not involve any human participants. An application for ethics waiver was made to the Human Research Ethics Committee of the University of the Witwatersrand and was approved (number R14/49).

Note that full information on the approval of the study protocol must also be provided in the manuscript.

Field-specific reporting

Please select the one below that is the best fit for your research. If you are not sure, read the appropriate sections before making your selection.

☒ Life sciences ☐ Behavioural & social sciences ☐ Ecological, evolutionary & environmental sciences

For a reference copy of the document with all sections, see nature.com/documents/nr-reporting-summary-flat.pdf

Life sciences study design

All studies must disclose on these points even when the disclosure is negative.

Sample size	A total of 325 samples were collected from 15 wastewater treatment plants. This was part of existing surveillance activity. There were no preset expectations or thresholds, so sample size calculation was not meaningful.
Data exclusions	No data was excluded form analysis
Replication	A positive control was included in each amplification and sequencing run to confirm the success of the procedure. All attempts of at replication were successful
Randomization	Randomization was not applicable in this study design since we did not perform experiments to evaluate the effect of specific treatments or differences between groups and all data was collected as part of ongoing pathogen surveillance efforts.
Blinding	Each sample was processed in the laboratory using the sample reference number.

Reporting for specific materials, systems and methods

We require information from authors about some types of materials, experimental systems and methods used in many studies. Here, indicate whether each material, system or method listed is relevant to your study. If you are not sure if a list item applies to your research, read the appropriate section before selecting a response.

Materials & experimental systems

n/a	Involved in the study
☒	☐ Antibodies
☒	☐ Eukaryotic cell lines
☒	☐ Palaeontology and archaeology
☒	☐ Animals and other organisms
☒	☐ Clinical data
☒	☐ Dual use research of concern
☒	☐ Plants

Methods

n/a	Involved in the study
☒	☐ ChIP-seq
☒	☐ Flow cytometry
☒	☐ MRI-based neuroimaging
